# Evolution of lysine acetylation in the RNA polymerase II C-terminal domain

**DOI:** 10.1186/s12862-015-0327-z

**Published:** 2015-03-10

**Authors:** Corinne N Simonti, Katherine S Pollard, Sebastian Schröder, Daniel He, Benoit G Bruneau, Melanie Ott, John A Capra

**Affiliations:** Center for Human Genetics Research, Vanderbilt University, Nashville, TN 37232 USA; Gladstone Institutes, University of California, San Francisco, San Francisco, CA 94158 USA; Department of Epidemiology & Biostatistics and Institute for Human Genetics, University of California, San Francisco, San Francisco, CA 94158 USA; Departments of Biological Sciences and Biomedical Informatics, Vanderbilt University, Nashville, TN 37232 USA

**Keywords:** RNA pol II C-terminal domain, Heptad repeats, RNA pol II acetylation, Posttranslational modification, Metazoa, Phylogenetics

## Abstract

**Background:**

RPB1, the largest subunit of RNA polymerase II, contains a highly modifiable C-terminal domain (CTD) that consists of variations of a consensus heptad repeat sequence (Y_1_S_2_P_3_T_4_S_5_P_6_S_7_). The consensus CTD repeat motif and tandem organization represent the ancestral state of eukaryotic RPB1, but across eukaryotes CTDs show considerable diversity in repeat organization and sequence content. These differences may reflect lineage-specific CTD functions mediated by protein interactions. Mammalian CTDs contain eight non-consensus repeats with a lysine in the seventh position (K_7_). Posttranslational acetylation of these sites was recently shown to be required for proper polymerase pausing and regulation of two growth factor-regulated genes.

**Results:**

To investigate the origins and function of RPB1 CTD acetylation (acRPB1), we computationally reconstructed the evolution of the CTD repeat sequence across eukaryotes and analyzed the evolution and function of genes dysregulated when acRPB1 is disrupted. Modeling the evolutionary dynamics of CTD repeat count and sequence content across diverse eukaryotes revealed an expansion of the CTD in the ancestors of Metazoa. The new CTD repeats introduced the potential for acRPB1 due to the appearance of distal repeats with lysine at position seven. This was followed by a further increase in the number of lysine-containing repeats in developmentally complex clades like Deuterostomia. Mouse genes enriched for acRPB1 occupancy at their promoters and genes with significant expression changes when acRPB1 is disrupted are enriched for several functions, such as growth factor response, gene regulation, cellular adhesion, and vascular development. Genes occupied and regulated by acRPB1 show significant enrichment for evolutionary origins in the early history of eukaryotes through early vertebrates.

**Conclusions:**

Our combined functional and evolutionary analyses show that RPB1 CTD acetylation was possible in the early history of animals, and that the K_7_ content of the CTD expanded in specific developmentally complex metazoan lineages. The functional analysis of genes regulated by acRPB1 highlight functions involved in the origin of and diversification of complex Metazoa. This suggests that acRPB1 may have played a role in the success of animals.

**Electronic supplementary material:**

The online version of this article (doi:10.1186/s12862-015-0327-z) contains supplementary material, which is available to authorized users.

## Background

Eukaryotic RNA polymerases evolved from a single ancestral enzyme into three structurally related RNA polymerase enzymes (I–III) with specialized functions in eukaryotes. RNA polymerase II generates all protein-coding mRNAs as well as a large number of non-coding microRNAs (miRNAs), small nuclear RNAs (snRNAs) and small nucleolar RNAs (snoRNAs). The enzyme is composed of 12 subunits (RPB1–12), five of which are shared among the three eukaryotic polymerase complexes. The largest subunit, called RPB1, is unique to RNA polymerase II and is involved in its catalytic activity.

The C-terminal domain (CTD) of RPB1 is essential for the proper regulation of RNA polymerase II [1]. The CTD consists of a largely unstructured, repetitive stretch of tandem heptad amino acid repeats with a consensus sequence of tyrosine-serine-proline-threonine-serine-proline-serine (Y_1_S_2_P_3_T_4_S_5_P_6_S_7_). The CTD is connected to the core polymerase enzyme via a flexible linker close to the RNA exit site [2] and contains a protein-protein interaction surface for cofactors involved in the regulation of transcription initiation, elongation and RNA processing—highly specialized functions that determine the speed and reliability of the polymerase enzyme traversing a gene during transcription [3,4].

In mammals, the CTD heptad repeats undergo a sequence of characteristic posttranslational modifications during transcription [5] and these modifications determine which cofactors can bind [6]. Phosphorylation of the S_5_ residue occurs when the polymerase binds to gene promoters, and transcription is initiated [7]. The phospho-S_2_ modification is a hallmark of elongating polymerase complexes and is maintained until transcription termination occurs [8]. Phosphorylation of S_7_ residues in linker-proximal heptad repeats is important for the transcription of snRNAs and facilitates the interaction of the RNA polymerase II complex with the Integrator complex at these genes [9,10]. Methylation of a single arginine residue plays a role in the production of snoRNAs as well as snRNAs and serves as a binding site for the tudor domain–containing protein TDRD3 [11]. Phosphorylation of T_4_ residues was shown to play a novel function in 3′-end processing of histone pre-mRNAs [12]. Phosphorylation of Y_1_ was coupled to antitermination of transcription [13], and was recently associated with promoters, enhancers, and degradation of RNA polymerase II [14,15]. However, in spite of our increasing knowledge of these marks, the full number and identity of repeats that undergo modification during transcription are unknown.

The CTD repeat motif and tandem repeat orientation are observed in most eukaryotic model organisms, and this configuration evolved early in the history of eukaryotes [16]. However, there is considerable diversity in the number and sequence of the repeats across eukaryotic clades, and the complexity of the CTD is roughly correlated with developmental complexity in animal, plant, and fungal multicellular lineages [16]. It has been proposed that this diversity in CTD sequences reflects functional constraint due to lineage-specific CTD–protein interactions [17]. In mammals, non-consensus repeats are required for the stability of the CTD [18]. However, deletion of most of the non-consensus repeats does not affect the housekeeping functions of mammalian cells, but it does impair activator-induced gene transcription [18,19].

A novel CTD modification of non-consensus repeats necessary for the proper regulation of polymerase pausing was recently discovered [20]. Lysine residues at the seventh position (K_7_) in non-consensus distal RPB1 CTD repeats are acetylated (acRPB1) in mouse and human, but not in yeast (*Saccharomyces cerevisiae*), where the repeats lack lysines. This modification, performed by the acetyltransferase (KAT) enzyme p300/KAT3B, which is absent in yeast, distinctly marks promoter-proximally paused polymerases. Disruption of this mechanism interfered with the expression of two growth-factor-induced genes regulated by polymerase pausing, but did not influence expression or polymerase occupancy at two non-paused genes [20].

Knowledge of the evolutionary origins of a gene or pathway can inform analysis of its functions [21,22]. Thus, we undertook an evolutionary and functional analysis of the RPB1 CTD sequence and the genes influenced by acRPB1. We found that the presence of multiple K_7_ residues, and thus the potential for RPB1 CTD acetylation, arose with animal multicellularity during an expansion in the overall number of CTD repeats in Metazoa. Our phylogenetic analysis further showed that p300/KAT3B, the acetyltransferase that modifies the RPB1 CTD, was present at the appearance K_7_-containing repeats. We then performed a genome-wide survey of acRPB1 occupancy and its influence on gene regulation in mice. Genes with enrichment for acRPB1 at their promoters and genes dysregulated when acRPB1 was disrupted were specifically enriched for functions in growth-factor signaling, cell adhesion, vascular development, and cell-cell interaction. In addition, the two sets of acRPB1 sensitive genes were enriched for evolutionary origins in early eukaryotes through the ancestor of Euteleostomi (bony vertebrates). Together, our functional and evolutionary results support a model in which K_7_-containing CTDs were selected for in the early history of animal multicellularity. Given the association between polymerase pausing and acRPB1, the potential to acetylate these residues may have enabled tighter control of gene expression as animals grew in complexity and diversified. Indeed, acRPB1 now influences the regulation of growth factor-target genes and genes involved in lineage-specific processes, such as cell adhesion and vasculature development, in mammals.

## Results

### The RPB1 CTD experienced an expansion in repeat number and lysine content in the early history of animals

**Figure 1:**
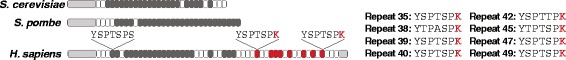
**The human RNA polymerase II subunit 1 (RPB1) C-terminal domain (CTD) contains more heptad repeats than the yeasts, and eight of its non-consensus distal repeats have a lysine residue.** In this schematic of the RPB1 CTD for two species of yeast and human, consensus heptad repeats (YSPTSPS) are colored dark gray; repeats with a lysine at position 7 are colored red; and all other non-consensus repeats are in white.

The RPB1 CTD has undergone considerable change during the evolution of eukaryotes [16]. While the consensus repeat sequence Y_1_S_2_P_3_T_4_S_5_P_6_S_7_ is conserved from yeast to mammals, the number of repeats varies: *S. cerevisiae* has only 26 repeats, while humans have 52 (Figure 1). Conserved heptad repeats are found in the linker-proximal part of the mammalian CTD, but the sequence of the distal heptad repeats, which are not present in yeast, diverge from this consensus sequence. Eight of the non-consensus repeats in human and mouse CTDs carry a lysine at position 7 (K_7_), rather than serine.

**Figure 2:**
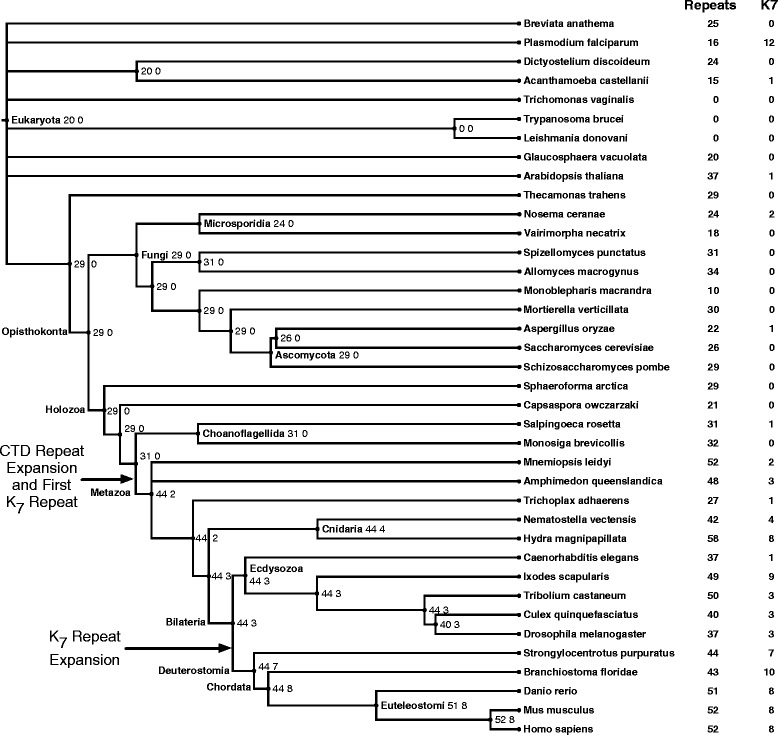
**Lysine-containing CTD repeats first appeared in Metazoa and increased in prevalence in the ancestor of Deuterostomia.** Phylogenetic tree of eukaryotic species considered in our analysis organized by approximate divergence estimates. For each species, the number of RPB1 CTD repeats and lysine-containing repeats are given. Ancestral counts were inferred for each internal node of the tree using symmetric Wagner parsimony. The number of CTD heptad repeats increased substantially in the ancestor of all Metazoa (31 to 44 repeats). This was accompanied by the appearance of repeats with lysine at position seven (K_7_) in ancestral Metazoa and an increase in K_7_ repeats in Deuterostomia (from 3 to 7 repeats).

To gain insight into the origins of mammalian K_7_-containing repeats, we modeled the evolutionary dynamics of CTD repeats in a phylogenetically diverse collection of eukaryotes with sequenced *RPB1* genes (Figure 2). Human, mouse, and zebrafish were selected as representative vertebrate species, based on their phylogenetic placement and sequence data quality. In addition to the three vertebrates, we also examined CTD amino acid sequences for 35 other eukaryotes, including worms, insects, fungi, plants, algae, and several recently sequenced early branching animals. For each species, we counted the number of CTD repeats overall, the number of consensus repeats, and the number of repeats with lysine residues (Figure 2; Additional file 1).

Ancestral state reconstruction using symmetric Wagner parsimony on the species phylogeny revealed an expansion of CTD repeats in the common ancestor of all Metazoa (Figure 2), as expected from previous studies [16,17]. We estimate that the ancestor of all Metazoa had 44 repeats, while the last common ancestor of Metazoa and their closest relatives, the choanoflagellates, had only 31 repeats. K_7_-containing repeats also first appeared consistently in the ancestor of Metazoa, and this was followed by an increase in the number of K_7_-containing repeats (from 3 to 7) in the last common ancestor of Deuterosomia. All the Cnidaria and Ecdysozoa examined have K_7_-containing repeats, but with the exception of the deer tick (*Ixodes scapularis*) and hydra (*Hydra magnipapillata*), the number is comparatively small (see Discussion). The plants, algae, and other eukaryotes we examined have half as many or fewer repeats as the Metazoa, and only one has more than two lysine-containing repeats: *Plasmodium falciparum,* a human pathogen. (See the Discussion and [23] for more on the evolution of the *Plasmodium falciparum* CTD.) Thus, we conclude that CTD repeat length markedly increased with the origin of animals, and the distal repeats gained lysine residues and expanded further in different animal lineages. These results are in agreement with an extensive study of CTD repeat number and content that appeared while this manuscript was in preparation [16].

**Figure 3:**
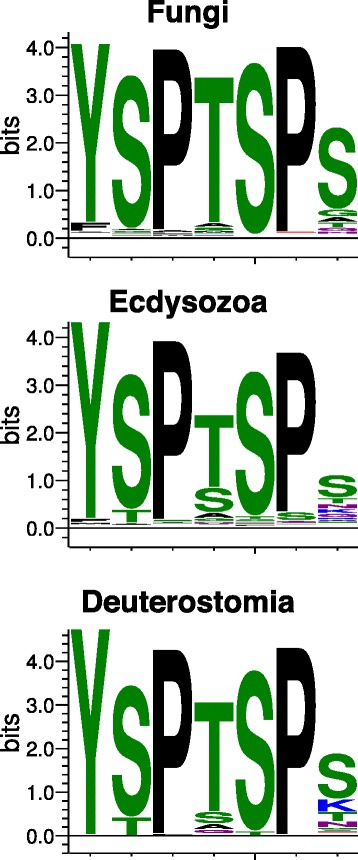
**Sequence logos summarizing repeat sequence variation in Fungi, Ecdysozoa, and Deuterostomia.** Sequence logos were generated from all heptad repeat sequences of relevant species using the LogOddsLogo tool [41]. Most repeat positions are strongly conserved, but different clades exhibit different variations. Lysine is particularly common at position seven in Deuterostomia. See Additional file 1 for RPB1 sequences for all species examined.

We found variations on the consensus CTD heptad repeat in all clades (Figure 3). Overall, the seventh position of the heptad repeat is the most variable, but different clades exhibit different patterns of variation. Lysines are common in the seventh position among the deuterostomes, and the repeat profiles for individual deuterostome species (excepting *Brachiostoma floridae*) are more similar to one another than for any other clade. They average only 3% difference between species. In Ecdysozoa, positions four, five, and six are more variable than in other clades, with serines and alanines common in position four. Fungal CTD repeats, with the exception of those in *Aspergillus oryzae* (Figure 3), largely exhibit the consensus sequence; however, they have more variation in the first position than other clades. We also note that some species have a small amount of additional non-repetitive sequence beyond the CTD repeats. CTD repeat counts and sequences for all species examined are given in Additional files 1 and 2, respectively.

### The acetyltransferase p300 was present for the metazoan increase in CTD repeat count and lysine content

The p300/KAT3B acetyltransferase was shown to efficiently target and acetylate the mammalian RPB1 CTD, while another KAT enzyme, PCAF/KAT2B, did not [20]. Recent analysis of the *Capsaspora owczarzaki* genome revealed that p300/KAT3B and its paralog, CBP/KAT3A, were present before the divergence of *Capsaspora* from choanoflagellates and metazoans [24]. To further refine this estimate, we searched for homologs in the additional early branching holozoan species considered in our CTD analysis and other eukaryotic genomes. All tested holozoans contain a gene with significant homology (all have BLAST E-values < 7E-155) to human/mouse p300 and CBP; thus, the ancestor of this gene was present for the metazoan expansion of the CTD and introduction of K_7_-containing repeats. Homologs of this family are present in plants [25], but we did not find clear homologs in the fungi. This suggests that the KAT3 family may be a more ancient eukaryotic family that has been lost in some lineages and maintained in others.

We also determined the phylogenetic age of 17 KAT enzymes with expressed in both mouse and humans [26] using ProteinHistorian [22], a web server that estimates protein ages, and found a wide range of predicted evolutionary ages (Additional file 3).

### Acetylation of RPB1 regulates many mammalian genes

Mutating the RPB1 CTD to prevent acetylation was shown to disrupt the expression of two immediate-early genes (*c-Fos* and *Egr2*) in the epidermal growth factor (EGF) and ERK/MAPK pathways with paused polymerases, but it did not influence the expression of several housekeeping genes [20]. To explore the functional relevance of acetylation of the RPB1 CTD genome-wide, we analyzed two sets of genes influenced, directly and indirectly, by acRPB1. First, we analyzed the total and acetylated RNA Pol II promoter occupancy in mouse embryonic stem cell (ESC) ChIP-Seq data from Schroeder et al. [20]. We found 10,078 genes with acetylated RNA Pol II occupancy at their promoters, and 707 genes with significant enrichment for acetylated RNA Pol II at their promoters over total RNA Pol II levels (Methods; Additional file 4). We will refer to these gene sets as “acRPB1 occupied” and “acRPB1 enriched,” respectively. Second, we expressed a mutated murine HA-tagged RPB1 in which all K_7_ residues were substituted by arginines (8KR) in mouse NIH/3 T3 fibroblasts (Methods; [20]). This mutation prevents acetylation while preserving the positive charge at these positions. Gene expression profiling using microarrays identified 1787 RNAs that were significantly (False Discovery Rate (FDR) < 0.05) up- or down-regulated in 8KR cells compared to wild type cells. We refer to these as “acRPB1 dysregulated genes”. In the following, we focus on the acRPB1 enriched genes, which reflect the direct influence of acRPB1, and the acRPB1 dysregulated genes, which summarize the broader downstream effects of RPB1 acetylation.

There was significant overlap between the acRPB1 enriched and dysregulated genes (83 genes; p < 0.0001, chi-squared test). The relatively modest magnitude of this overlap likely reflects the different origins of the gene sets. The ChIP-Seq was performed on ESCs, while the microarrays are from fibroblasts. AcRPB1 is likely involved in many context-dependent responses in different cells, and a gene with enrichment for acRPB1 at its promoter in one context is not necessarily dysregulated in all contexts if the ability to acetylate RPB1 is lost. It also suggests that some genes dysregulated in the disruption of acRPB1 may not be directly regulated by acRPB1. However, it is also possible that some differences in the gene sets result from technical artifacts due to the different experimental techniques used to define them.

### Genes influenced by acetylation of RPB1 are enriched for functions in growth-factor response, cell adhesion, regulation of gene expression, and vasculature development

We used DAVID [27,28] to calculate Gene Ontology Biological Process (GO BP) annotations and KEGG pathways enriched among acRPB1 enriched (Table 1) and dysregulated genes (Table 2). To account for the many annotations tested, we considered tests with expected FDR < 0.05 significant. The acRPB1 enriched genes are annotated with general regulatory GO BP functions more often than expected by chance; for example, protein amino acid phosphorylation (FDR = 0.01) and negative regulation of gene expression (FDR = 0.02) are both significantly enriched. The dysregulated genes are enriched in GO BP functions in cell adhesion (FDR = 6.6E-9), vasculature development (FDR = 7.3E-4), and blood vessel development (FDR = 8.3E-4). The top KEGG pathway enrichments for both gene sets were largely consistent with the GO BP analysis, though some did not maintain significance after FDR-based multiple test correction. Within KEGG, the acRPB1 enriched genes had nominally significant overlap (uncorrected p < 0.05) with genes involved in several developmental signaling pathways, focal adhesion, and actin cytoskeleton regulation (Additional file 5). Similarly, the dysregulated genes showed enrichment for the focal adhesion (FDR = 0.001) and extracellular matrix receptor interaction (FDR = 0.02) pathways, as well as weaker associations with pathways involved in actin cytoskeleton regulation, axon guidance, and several signaling and metabolic pathways (Table 2; Additional file 5). Thus, the direct and indirect targets of acRPB1 appear to be involved preferentially in regulation (both signaling and transcription), cell adhesion, and vasculature development.

**Table 1:** **Functional annotations and pathways enriched among acRPB1 enriched genes**

**Functional Annotation**	**Number of genes**	**Fold enrichment**	**Raw p-value**	**FDR**
Protein amino acid phosphorylation (GO BP)	42	2.1	7.09E-06	0.01
Neg. regulation of gene expression (GO BP)	34	2.2	1.15E-05	0.02
Neg. regulation of macromol. biosynthetic process (GO BP)	34	2.1	2.89E-05	0.05

**Table 2:** **Functional annotations and pathways enriched among acRPB1 dysregulated genes**

**Functional Annotation**	**Number of genes**	**Fold enrichment**	**Raw ** **p-value**	**FDR**
Cell adhesion (GO BP)	105	2.0	3.6E-12	6.6E-09
Vasculature development (GO BP)	51	2.1	4.0E-07	7.3E-04
Blood vessel development (GO BP)	50	2.1	4.6E-07	8.3E-04
Focal adhesion (KEGG)	42	2.2	1.1E-06	0.001
ECM-receptor interaction (KEGG)	22	2.8	1.5E-05	0.019
Blood vessel morphogenesis (GO BP)	40	2.1	1.1E-05	0.019

The disruption of acRPB1 was previously shown to abolish expression of two epidermal growth-factor-induced genes, *c-Fos* and *Egr2*, in mouse fibroblasts [20]. In order to further explore the connection between EGF-induced genes and RPB1 CTD acetylation, we analyzed the expression and promoter acRPB1 occupancy of several curated sets of growth factor responsive genes. First, we identified 49 mouse homologs of human genes whose transcription is directly induced by EGF signaling (Additional file 6) [29]. Overall, EGF-induced genes overlap significantly with acRPB1 enriched genes (5 of 48 with promoter occupancy data, p = 0.001, Yates’ chi-squared test), as well as the dysregulated genes (10 of 49; p = 0.001). The association between acRPB1 and EGF became even stronger when we considered promoter acRPB1 occupancy (regardless of whether it was significantly higher than total RPB1 occupancy): 73% (35 of 48) of EGF genes had acRPB1 occupancy (p = 1E-8). Similarly, the number of EGF genes nominally dysregulated (p < 0.05) was also significant (19 of 49; p = 0.003).

Given the association between acRPB1 and EGF-induced genes, we investigated whether acRPB1 regulated genes induced by other growth factors by analyzing 100 genes induced by platelet-derived growth factor (PDGF; Additional file 6) [30]. There was a similar enrichment for PDGF-induced genes among acRPB1 occupied (65 of 100; p = 0) and dysregulated (23 of 100; p = 8E-8) genes, but not among acRPB1 enriched genes (2 of 100; p = 0.906). Thus, genes induced by both growth factors are regulated by acetylation of the RPB1 CTD, though perhaps mostly indirectly in the case of PDGF. Supporting this conclusion, EGF-induced genes were more strongly enriched among acRPB1-enriched genes than PDGF-induced genes (p = 0.008, Fisher’s exact test), but both growth factors were similarly represented among dysregulated genes (p = 0.381). Fifteen genes were present in both the EGF- and PDGF-induced sets; the above results were similar when these genes were not considered in the analysis.

Growth factor-induced genes can be split into two categories: immediate-early genes (IEGs) and delayed primary response genes (DPRGs), which are transcribed after the IEGs, but before secondary response genes [30]. These two classes are relevant, because their transcription occurs before *de novo* protein synthesis, and thus they reflect the direct effects of growth factor signaling versus those further downstream. Regardless of the growth factor, both IEGs and DPRGs were enriched among acRPB1-occupied and -dysregulated genes, but the effect was stronger for IEGs than DPRGs (Fisher’s exact test p = 0.047) supporting the hypothesis that acRPB1 targets many IEGs.

### Many genes regulated by acetylation of RPB1 originated early in the evolution of eukaryotes and animals

**Figure 4:**
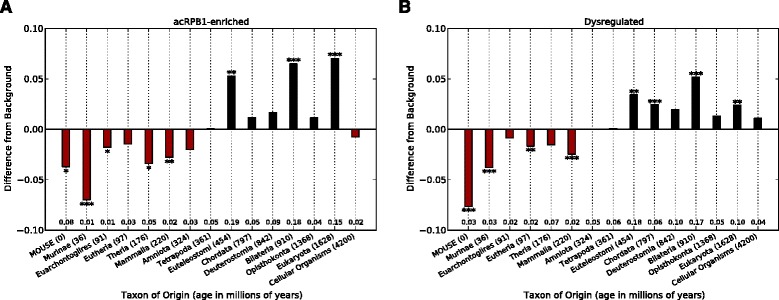
**Genes regulated by acRPB1 are significantly enriched for evolutionary origins from early eukaryotes through early animals.** ProteinHistorian analysis of the evolutionary origins of acRPB1 enriched genes **(A)** and acRPB1 dysregulated genes **(B)** compared with relevant background gene sets. Each bar gives the difference between the proportion of genes of interest and background genes originating in the last common ancestor of a given taxon (* = p < 0.05, ** = p < 0.01, *** = p < 0.001, all Bonferroni corrected). The proportion of genes of interest with origins in each taxon is given along the x-axis. For example, 18% of the acRPB1 enriched genes likely appeared between the origin of Opisthokonta and Bilateria, and this is significantly more (~7%, p < 0.001) than expected from the background of all genes with occupancy data.

Evolutionary analysis by ProteinHistorian showed that acRPB1-enriched genes had a significantly different age distribution from all genes with occupancy data (Figure 4A, p ≈ 0, Mann–Whitney *U* test). The acRPB1-enriched genes were significantly enriched for origins on the branch from Opisthokonta to Bilateria (18.3% vs. 11.8%, p < 0.001), the branch from Chordata to Euteleostomi (19.2% vs. 13.9%, p < 0.01), and the ancestral eukaryote branch (14.8% vs. 7.7%, p < 0.001). (Note that due to the species present in the ProteinHistorian database, the resolution of the protein age analysis is not as high as the CTD repeat analysis.) Comparing the evolutionary origins of dysregulated genes to the background of all genes on the array revealed a similar age pattern (Figure 4B; p = 1.4E-52). In particular, the dysregulated genes were enriched for origins in the ancestral eukaryote, the early history of animals (Opisthokonta to Bilateria branch), and shortly thereafter on the Deuterostomia to Chordata and Chordata to Euteleostomi branches (p < 0.001 for each). Both gene sets showed consistent depletion of genes born after the origin of amniotes. Thus, two independent ways of defining genes influenced by acRPB1 underscore a potential role for K_7_ acetylation in regulating RNA polymerase II function at genes that were present as animal multicellularity developed and diversified (Figure 4).

**Figure 5:**
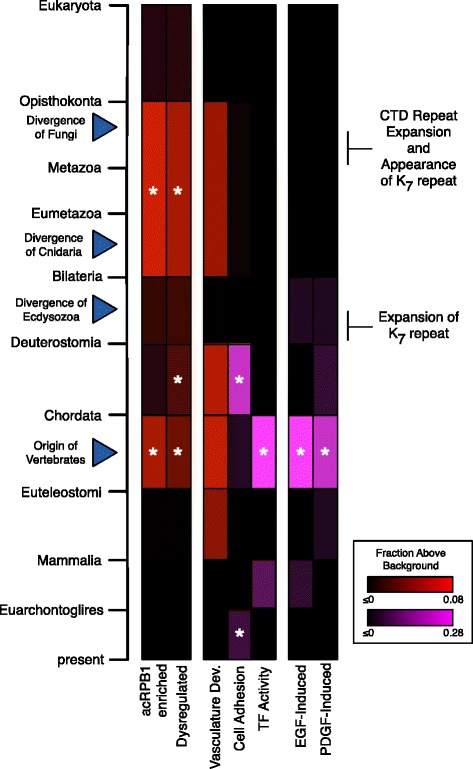
**Summary of the origins of subsets of genes regulated by acRPB1 with respect to relevant evolutionary events.** The period between K_7_ repeat expansion and the diversification of vertebrates, shows particular enrichment for the origin of acRPB1 regulated genes and genes with functions relevant to animals. Each column in the heat map represents the evolutionary origin distribution of a set of genes. Color intensity reflects the magnitude of increase over background in each evolutionary window. Due to the large difference for some gene sets, two scales were necessary (red and purple). Asterisks indicate significant increase over background (Bonferroni-corrected p < 0.05). Relevant evolutionary events are identified with blue triangles. For reference, the first two columns represent the acRPB1 enriched and dysregulated origin distributions (Figure 4). The remaining columns give the origin distributions for functional subsets of genes enriched among acRPB1 sensitive genes.

Many genes involved in functions enriched among the acRPB1-regulated genes have origins close to the origin of K_7_ repeat-containing CTDs or later during the origin and radiation of vertebrates (Figure 5). The window between the K_7_ repeat expansion and the diversification of vertebrates is enriched with the origin of acRPB1-regulated genes. For example, the EGF-induced genes are significantly enriched for origins on the branch leading to Euteleostomi (p < 0.0001), as are the PDGF-induced genes (p < 0.0001) (Figure 5). Consistent with this pattern, twelve of the 17 (71%) genes in the EGF signal transduction pathway (BIOCARTA), which are necessary for EGF induction, were born between the origin of Bilateria and Euteleostomi; three were present in the last common ancestor of eukaryotes (e.g., MAPK), and only one appeared after the Euteleostomi (Additional file 6). Thus, this pathway’s evolutionary history mirrors that of the genes influenced by acRPB1 (Figure 4).

## Discussion

### AcRPB1 likely first occurred early in the development of animals

Our integrated evolutionary and functional analyses of RPB1, its modifiers, regulators, and targets suggest that the potential for acRPB1 was present in early animals. While we cannot definitively establish the timing of the first acRPB1 event, several lines of evidence suggest that acRPB1 began to occur soon after K_7_-containing heptad repeats appeared during the increase in RPB1 CTD repeat length in the metazoan ancestor. First, elevated repeat count and K_7_-containing repeats are present in nearly all Metazoa. Second, K_7_-containing repeats are rare outside of Metazoa. Third, the ancestor of the p300 KAT enzyme, which acetylates CTD K_7_ residues, was present when the CTD expanded. Fourth, the genes bound and regulated by acRPB1 are enriched for evolutionary origins before and during the appearance and diversification of animals and depleted for origins more recent than the last common ancestor of all bony vertebrates (Euteleostomi). Finally, the functions enriched among genes regulated by acRPB1 suggest involvement in traits essential to the origin and development of complex animal multicellularity.

### AcRPB1 may have served as a basis for establishing the regulation of some functions essential to complex multicellular animals

Transitions to multicellular lifestyles require organisms to perform a range of new functions that facilitate cell adhesion, cell-cell signalling, and context-dependent gene expression [31,32]. We found enrichment for functions in cell adhesion, extracellular matrix signaling, gene regulation, and phosphorylation among genes influenced by acRPB1 (Tables 1 and 2). This suggests a connection between new regulatory mechanisms, such as polymerase pausing, enabled by acRPB1 and the development of animal multicellularity. In addition, the enrichment for vascular development among the genes sensitive to acRPB1 supports the involvement of acRPB1 in the later development and regulation of animal body plan complexity. Indeed, intercellular regulation of proliferation, differentiation, and migration by paracrine factors such as EGF is a unique feature of higher eukaryotes and lies at the root of body complexity in animals [33]. Our analyses show that proper regulation of genes induced by two growth factors, EGF and PDGF, is dependent on the presence of K_7_ residues in the CTD. While our study explicitly provides evidence for the importance of K_7_ residues in EGF- and PDGF-induced transcription, it also suggests that other signal transduction pathways in higher eukaryotes may rely on K_7_ residues for proper gene activation.

### What is the role of acRPB1 in different animal lineages?

The eukaryotic RPB1 CTD experiences posttranslational phosphorylation and methylation modifications that are essential for proper regulation of its activity. The initial expansion in CTD repeat number during the origin of Metazoa likely increased the potential for these RPB1 modifications. Our work suggests that acRPB1 appeared soon after this initial expansion. The integration of K_7_ residues into the RPB1 heptad repeat appears to predate the divergence of the Cnidaria, Ecdysozoa, and Deuterostomia; however, these clades exhibit differences in the number and type of repeats. Deuterostomes exhibit dramatic and consistent high K_7_ count (Figure 2). Nonetheless, the presence of at least one K_7_-containing repeat is conserved in all species analyzed in these clades, and some species (*H. vulgaris* and *I. scapularis*) have high K_7_ counts. Given the role of acRPB1 in promoter-proximal polymerase pausing [20] and the importance of pausing in non-deuterostome species, such as *Drosophila melanogaster* [34], it is possible that acRPB1 occurs commonly in these species in spite of their lower K_7_ content. Indeed, a single K_7_-containing repeat in a modified mouse CTD was sufficient to produce detectable acetylation *in vitro* [20]. Most pausing research has been performed in human, mouse, or fly, and the full phylogenetic range of polymerase pausing is not known. However, it appears that pausing is rare in *Caenorhabditis elegans* [35], a species with only one K_7_ repeat, but systematic studies of pausing and acRPB1 in a more diverse array of species are necessary to resolve this question.

### AcRPB1 may be associated with developmental complexity in different eukaryotic lineages

Recent work has established the plasticity of the RPB1 CTD across eukaryotes and suggested that lineage-specific CTD modifications are associated with increased complexity in different multicellular lineages [16]. While our work does not prove the involvement of acRPB1 in the development of animal multicellularity, it suggests that this novel regulatory mechanism may have played a role in the establishment of the complex gene regulatory programs necessary for the proper cooperation of different cells within complex multicellular animals, in addition to known expansions and rearrangements of gene families [36].

The presence of a non-consensus K_7_ repeat in many plant species [16] suggests the possibility that acRPB1 could have evolved in parallel in another developmentally complex multicellular lineage. However, it is not known whether plant K_7_ repeats are acetylated.

### Why are many K_7_-containing repeats seen in some species of malaria?

K_7_-containing repeats are rare outside of animals, yet in agreement with previous studies, we see many K_7_ repeats in *P. falciparum*, a parasite that causes malaria in humans (Figure 2). In general, *Plasmodium* species have very short repeat regions with high variability between and within species [16,23]. Primate-infecting *Plasmodium* species have many K_7_ repeats, yet this is not true of those that infect other mammals, like rodents, and birds. Analysis of the CTDs of many *Plasmodium* species suggests that expansion of K_7_ repeats in the CTD occurred twice in parallel in different lineages of primate parasites; however, the forces driving these independent primate-specific expansions are not known. The establishment of the acetylation of the CTD [20] and its importance in regulating many ancient genes suggests that CTD acetylation may be involved. However, since acRPB1 occurs in mice, the presence of K_7_ repeats and acRPB1 in the host does not necessarily result in high K_7_ content in the *Plasmodium* CTD. Horizontal transfer of epigenetic regulators, like the Set2 and Set8 methyltransferase domains, occurred in the ancestor of Apicomplexans and has been linked to their transition to parasitism [37]. The Set2 domain interacts with the CTD, but it is absent in rodent infecting *Plasmodium* species. This suggests that there may be differences in the epigenetic methylation and acetylation of the CTD in *Plasmodium* species infecting different mammals, but the nature and effects of these modifications remain uncertain.

## Conclusions

In this work, we trace the evolutionary origin of K_7_-containing RPB1 CTD repeats to the early history of animals. Our integrated evolutionary and functional analyses suggest that the potential for acRPB1 was present at this time and suggest, due to the conserved increased CTD repeat count and K_7_ content, that acRPB1 is a common regulatory mechanism in many animals. Most studies of the genetic changes involved in transitions to animal multicellularity have implicated expansions or rearrangements of gene families [36]. While our work does not prove the involvement of acRPB1 in this transition, it suggests that this novel regulatory mechanism may have provided a foundation on which gene regulatory programs involved in the proper function and cooperation of different cells within complex multicellular animals could be built.

## Methods

### Phylogenetic analysis

We identified species with sequenced RPB1 genes using BLAST searches and previous studies of RPB1 [38]. We confirmed that all sequenced vertebrates have the same pattern of repeats as human and selected human, mouse, and zebrafish (*Danio rerio*) as representative vertebrate species, based on their phylogenetic placement and sequence data quality. Removal of other vertebrate sequences avoids problems with oversampling, as well as problems associated with poor quality or missing data in some species. In addition to the three vertebrates, we included RPB1 amino acid sequences for 35 other eukaryotes, including worms, insects, plants, algae, amoebas, fungi, and several recently sequenced, early branching animals. For each species’ RPB1 sequence, we manually counted the number of CTD heptad repeats and the number of CTD repeats with lysine residues (Additional file 1). To be classified as an acceptable heptad, three of the seven amino acids had to match the consensus sequence or common alterations seen in multiple species.

We modified a species tree downloaded from the NCBI Taxonomy Database to reflect recent research on the evolutionary relationships between the clades considered and the approximate timing of divergence events [36,39]. To model losses and gains of CTD repeats and lysine content within each ancestral clade, we used Wagner parsimony (with an equal weight for gains and losses) as implemented in the Count program for analysis of numerical observations on a phylogeny [40].

Estimates of the phylogenetic age of human and mouse genes were made with ProteinHistorian using asymmetric Wagner parsimony on the PPOD-PANTHER protein-family database [22]. ProteinHistorian was also used to identify significant differences in the distribution of gene ages between gene sets of interest, such as those occupied by different polymerase forms or in different functional classes.

### Sequence logo generation

Sequence logos for clades were created using the online tool LogOddsLogo [41]. In generation of the summary logos for clades, all heptad repeats from the specified species were input into the generator.

### Identification of acRPB1 occupied and enriched genes

To determine promoter RPB1 occupancy genome-wide, we used ChIP-Seq data collected with antibodies for total RPB1 and acetylated RPB1 in mouse embryonic stem cells from [20]. Following their definitions, we considered a gene to be “RPB1 occupied” if it had total RPB1 signal at its promoter (2 kilobases upstream of the transcription start site) greater than twice the input signal at the promoter (total/input > 2). Similarly, we defined “acRPB1 occupied” genes as those with acRPB1 occupancy twice that of the promoter input signal (ac/input > 2). Since the amount of acRPB1 at a promoter strongly correlated with the amount of RPB1 [20], we defined a set of “acRPB1 enriched” genes that were RPB1 occupied and had acRPB1 promoter occupancy at least twice the RPB1 occupancy (total/input > 2 and ac/total > 2). The genes in each set are listed in Additional file 4.

### Identification of genes dysregulated with the disruption acRPB1

We used a HA-tagged mouse RPB1 construct in which all K_7_ residues were substituted with arginines (8KR) [20]. This mutation resembles unacetylated lysines by conserving the positive charge at these positions, but preventing acetylation. To examine the potential functions of K_7_ acetylation in regulating gene expression, we stably expressed wildtype or 8KR HA-RPB1 in murine NIH/3 T3 fibroblasts and cultured these cells in media containing α-amanitin. Both were expressed at equivalent levels, but acetylation was present only in wildtype, and not mutant HA-RPB1. We then performed gene expression profiling using the Affymetrix Mouse Gene 1.0 ST microarray with three biological replicates. Array values were normalized and log_2_ scaled. For statistical analyses, we removed all array probe sets in which no experimental groups had an average log_2_ intensity greater than 3.0. This is a standard cutoff, below which expression is indistinguishable from background noise. Linear models were fit for each gene with the Bioconductor “limma” package in R [42,43]. Moderated t-statistics, fold-change and the associated p-values were calculated for each gene. To account for the fact that thousands of genes were tested, we controlled the false discovery rate (FDR) using the Benjamini-Hochberg method [44]. Genes with p-values corresponding to expected FDRs of 0.05 or less were considered dysregulated compared to wildtype. These genes are listed in Additional file 4. The raw array data are available in the Gene Expression Omnibus (GSE66088).

### Functional annotation of genes

We used the online functional annotation tool DAVID [27,28] to calculate KEGG pathway and gene ontology functional annotation enrichment for each set of genes of interest. EGF-induced and PDGF-induced genes were taken from [29] and [30]. The human gene identifiers from these studies were mapped to their mouse homologs using the HGND database.

### Availability of supporting data

The data sets supporting the results of this article are available as additional files. The microarray data we collected are available from the Gene Expression Omnibus (accession number GSE66088), and the previously collected ChIP-Seq data [20] are available from the Sequence Read Archive (accession number SRX338012).

## Additional files

Additional file 1:
**RPB1 CTD repeat counts for all species considered.**
Additional file 2:
**RPB1 CTD sequences for all species considered.**
Additional file 3:
**Taxa of origin for human/mouse acetyltransferase genes as predicted by ProteinHistorian.**
Additional file 4:
**Lists of acRPB1 occupied, acRPB1 enriched, and dysregulated genes.**
Additional file 5:
**GO Biological Process and KEGG pathway enrichment among acRBP1-enriched and dysregulated genes.**
Additional file 6:
**Lists of EGF and PDGF genes.**
